# Psychiatric morbidity in adult Kashmiri migrants living in a migrant camp at Jammu

**DOI:** 10.4103/0019-5545.64597

**Published:** 2010

**Authors:** Rakesh Banal, Jagdish Thappa, H. U. Shah, Arshid Hussain, Abhishek Chowhan, Harneet Kaur, Mala Bharti, Sushant Thappa

**Affiliations:** 1 State Health Services, Government Medical College, Jammu & Kashmir, India; 2 State Health Services, Government Medical College, Srinagar, Jammu & Kashmir, India

**Keywords:** Migrants, psychiatric morbidity, refugees, trauma

## Abstract

**Background:**

There are 14.9 million refugees and 22 million internally displaced persons in the world. The clinical and research literature shows a significant degree of psychological stress among refugees with relatively high levels of physical and psychological dysfunction in them.

**Aims:**

To determine the prevalence of various psychiatric disorders among Kashmiri migrants settled in a migrant camp at Jammu

**Materials and Methods:**

This study was conducted on adults of Kashmiri migrant families residing in Muthi camp at Jammu. Three hundred families (150 each from two camps) were taken up for the study. Psychopathology was measured using Mini International Neuropsychiatry Interview Schedule (MINI). The data was categorized according to age, sex, education. The data was analyzed using Chi-square test with Yate’s correction wherever required. *P*-value less than 0.05 was taken as significant.

**Results:**

Psychiatric morbidity was more in migrant population 33.66% (n=208) than in controls 26% (n=52) with major depressive episode being the most common diagnosis

**Conclusions:**

Depression, post-traumatic stress disorders (PTSD) and generalized anxiety disorders (GAD) were statistically more prevalent among migrants than in controls.

## INRODUCTION

It is estimated that 1% of world’s population has been displaced either from their home or from their home country.[1] A report of the United States committee of refugees published in the year 2002 estimates that there are 14.9 million refugees and 22 million internally displaced persons in the world.[2]

Refugees are clearly defined by the international legal conventions and, therefore, are entitled to protection and assistance by the United Nations High Commission for refugees. In contrast, persons who flee their homes for the same reasons as for refugees, but who remain inside their own countries, enjoy no such rights. These "internally displaced" persons are in a particularly precarious situation because they are often beyond the reach of international agencies, which rely on the cooperation of national governments to deliver relief aid.[2]

Internal displaced persons can be seen as different from refugees in the sense that they are not displaced to a different country but still similarities exists between the two because of significant "cultural shock" and predisplacement isolation. Refugees or internally displaced persons leave their homeland and culture with little hope of return. "Culture shock" is thus overwhelming, especially for the unrelenting older generations, with less ability to adapt. Displacement is often accompanied by physical and/or psychological trauma. Even without the accompanying trauma, displacement is a wrenching event.

The clinical and research literature shows a significant degree of psychological stress among refugees with relatively high levels of physical and psychological dysfunction during the first two years of resettlement. After a period of three years, there was some improvement and increased adaptability, but their serious and pervasive adjustment problems, such as high levels of somatization, depression and post traumatic stress disorder (PTSD), continued to affect some sectors of the refugee population. These symptoms have even been noted many years after resettlement.[3,4]

Refugee’s health epidemiology is very complex and wide ranging, especially given the diversity in background socio economic status, ethnicity, geographical, and the like. Despite these dissimilarities, however, most refugees also share common experiences. For example, they have undergone physical and psychological hardships in the period prior to resettlement.[5] The first epidemiological evaluation of psychiatric disorders in displaced persons dates back to more than 70 years ago.[6] Epidemiological studies amongst displaced and war-affected populations have yielded high rates of mental disorders, especially post-traumatic stress and depression symptoms.[6–8] However, there is a dearth of research on whether displaced persons remain at increased risk of mental disorders in the long term.[10] Two relevant studies undertaken amongst Indo-Chinese population present somewhat discrepant findings. Westermeyer *et al*[11] reported that 90% of Hmong refugees were depressed 1.5 years post-migration. By three to four years, this rate had fallen to 19%, remaining roughly stable at 10 years (21%), a prevalence that remained higher than amongst the US host population. Beiser and Feng[8] on the contrary, showed a gradual but consistent reduction in prevalence of major depression from 65% at one to two years following migration, to 4.4% at three to four years and 2.3% at 10 years, a rate that was lower than amongst the host population. However, both studies suggest initial high rates of mental disorders among the refugees which improved over time. Still, most of the available literature on refugees’ mental health is limited to refugees living in the West[12] despite the fact that majority of the refugees live in low income countries like India.

Kashmir is known for its natural beauty for centuries. However, the 17-year-old armed conflict in Kashmir has disturbed various dimensions of human life: personal, social and political among others. The total population of Jammu and Kashmir, as per a report published in the year 2001, was 1, 00, 69,917; out of this, Hindu population in the state was 19, 30,448.[13] Prior to migration, Kashmiri Pandits were concentrated in Kashmir valley. They constituted nearly 8% of Kashmir’s population. In the year 1990, migration took place throughout the valley. Prevailing situation at that time forced Kashmiri Pandit families to move out of their motherland, within a short span of one week to fortnight.[14]

According to estimates by the Government of India, 56246 families have migrated from the valley since 1990. Of these, 34305 families stay in Jammu, 19338 families in Delhi and 2603 in other states.[15] While majority of the migrants are living under their own arrangements with friends, relatives etc, and in hired accommodation, 4778 families in Jammu and 238 families in Delhi are staying in relief camps.

In Jammu, the relief camps are at Battal Ballian (with 385 families), Jheri (265 families), Mishriwala (681 families), Muthi-I (490 families), Muthi-II (486 families), Purkhoo-I (404 families), Purkhoo-II (250 families), Purkhoo-III (916 families), Nagrota-I (402 families), Nagrota-II (97 families), Nagrota-III (104 families). In Delhi, 238 families live in various camps at Hauz Rani, Bapu Dham, Moti Nagar, Palika Bazar and Baljeet Nagar.[15]

The trauma of forced exodus and the exposure to an alien and hostile environment are further compounded by the problems of acclimatization; lack of basic amenities like drinking water, drainage and sewerage, absence of proper lavatory facilities, poor housing, over crowding, extremes of climate, lack of healthcare facilities and joblessness. All these have led to many psychological and behavioral disturbances in them.[16] But to the best of our knowledge, there is no study which has assessed the magnitude of these psychological problems, which manifest in the form of various psychiatric disorders. This study aims to determine the prevalence of various psychiatric disorders among Kashmiri migrants settled in a migrant camp at Jammu.

## METHODOLOGY

The study was conducted on adults of Kashmiri families residing in Muthi camp at Jammu. Muthi camp is located at about eight Kilometers (Kms) from Jammu city. Of the 996 Kashmiri families residing in Muthi camp, 300 families (150 families each from Muthi Camp I and Muthi Camp II) were taken up for the study. Controls for the study were taken from Muthi village of Jammu. This village is located around 2 Kms from the camp. A total of about 100 families were taken up for the study. In each phase, every third house was selected for the study for both the groups, till sample size was complete. Two adults, one male and one female, were selected from each family from the list of all adult family members. Controls, as far as possible, were matched for age and sex. As no psychiatric facilities were available in the camps and in Muthi village, persons who were diagnosed to have a psychiatric diagnosis were asked to contact Government Psychiatry Diseases Hospital, Jammu, located about 6 Kms from the camp.

To be included in the study, the subjects were required to be Kashmiri migrants (not applicable to controls) and be in the age group of 15 – 65 years. Subjects who did not consent, were less than 15 years or more than 65 years of age, had diagnosis of dementia or any other organic brain disease or had major psychiatric illness diagnosed before migration (not applicable to controls) were excluded. Psychopathology was measured using Mini International Neuropsychiatry Interview Schedule (MINI).

The data was categorized according to age, sex, education etc. The data was analyzed using Chi-square test with Yate’s correction wherever required. *P*-value of less than 0.05 was taken as significant.

## RESULTS

### Socio demography and psychatric morbidity

The maximum number of migrants 29% (n = 174) belonged to 35 – 44 years age group, while maximum number of controls 27% (n = 54) belonged to 55 – 65 years age group. The presence of psychiatric disorders was seen mostly (41.5%, n = 59) in 45 – 54 years age group, in the case of migrants; it was most common (40%, n = 10) in the 25 – 34 years age group, in the case of controls.

The number of males and females taken for the study was equal. In case of migrants the presence of psychiatric disorders in females was 37.3% (n = 112) which was greater as compared to males in whom it was 32% (n = 96). While in case of controls the presence of psychiatric disorder in females was 22% (n = 22) which was less as compared to males in whom it was 30% (n = 30). However, the differences were statistically not significant. Fifty seven per cent (n = 342) of the migrants were undergraduates followed by illiterates 36% (n = 216), graduates 36.3% (n= 38) and post graduates 0.6% (n = 4). The presence of psychiatric disorders was highest in undergraduates 38.39% (n =132). Amongst controls 51% (n = 102) were undergraduates followed by illiterates 39% (n =78), graduates 9.5% (n =19) and post graduates 0.5% (n = 1). Again, the highest psychiatric morbidity was seen in undergraduates 28.43% (n = 29). Majority of the migrants comprised of housewives 43.16% (n = 256), followed by the self employed 32.83% (n = 197), government-employed 11.33% (n = 68), pensioner 8% (n = 48) and unemployed 4.66% (n = 28). The presence of psychiatric disorders was highest in unemployed 46.42% (n = 13) and least in self employed 28.42% (n = 56). Majority of the controls comprised of housewives 44.5% (n = 89) followed by self employed 21.5% (n = 43), Government employed 17.5% (n=35), unemployed 10.5% (n = 21) and pensioner 6% (n = 12). The presence of psychiatric disorders was highest in unemployed 38.09% (n = 8) and least in case of Government employed 22.85% (n = 8)

### Psychiatric morbidity

Major depressive episode (MDE) was the most common psychiatric disorder in the case of migrants 21.5% (n = 129), followed by Generalized anxiety disorder (GAD) current 13.8% (n = 83) and PTSD 6.83% (n= 41). MDE was also the most common psychiatric disorder in case of controls 13.0% (n = 26) followed by alcohol dependence current 9.5% (n = 19) and GAD 7.5% (n=15) [Table 1].

**Table 1:** Psychiatric disorders among migrants and controls

Module	Psychiatric disorder	Psychiatric disorder present in migrants in %age (n).	Psychiatric disorder present in controls in %age (n).	X^2 (df=1)^
A	Major depressive episode	21.5 (129)	13.0 (26)	4.41*
	Major depressive episode	2.16 (13)	2.0 (4)	
	current			
	Major depressive episode	19.33 (116)	11.0 (22)	
	recurrent			
B	Dysthymia	2.33 (14)	2.5 (5)	0.018
C	Suicide risk current	5.66 (34)	2.5 (5)	2.36
D	(Hypo) Mania episode	3.0 (18)	2.0 (4)	0.23
	Hypo manic episode	0.83 (5)	0.5 (1)	
	current			
	Hypo manic episode past	0.33 (2)	0.0 (0)	
	Manic episode current	1.5 (9)	1.0 (2)	
	Manic episode past	0.33 (2)	0.5 (1)	
E	Panic disorder current	2.0 (12)	2.0 (4)	0.085
F	Agoraphobia current	3.66 (22)	3.0 (6)	0.043
G	Social phobia current	1.83 (11)	2.5 (5)	0.078
H	Obsessive compulsive	2.5 (15)	2.0 (4)	0.015
	disorder current			
I	PTSD current	6.83 (41)	2.5 (5)	3.99*
J	Alcohol dependence	3.5 (21)	9.5 (19)	8.8**
	current			
K	Substance dependence	0.83 (5)	1.0 (2)	0.048
	(non alcohol) current			
L	Psychotic disorder	0.66 (4)	1.0 (2)	0.0
	Psychotic disorder current	0.5 (3)	0.5 (1)	
	Psychotic disorder lifetime	0.16 (1)	0.5 (1)	
M	Anorexia nervosa current	0	0	0
N	Bulimia nervosa current	0	0	0
O	G.A.D current	13.8 (83)	7.5 (15)	3.98*

A large number of migrants and controls had more than one diagnosis, signifying co morbidity

* *P*<0.05

** P<0.001

## DISCUSSION

Our study was community-based, conducted on 300 Kashmiri migrant families. The prevalence of psychiatric disorders was maximum 41.5% (n=59) in the 45-54 years age group. Similar results were seen by Kristine Sandquist *et al*.[17] in their study on 163 female Bosnian refugees, in which they used Hopkins symptom check list (HSCL-25) and post-traumatic stress syndrome (PTSS-10) screen to measure the psychopathology. While Marshall *et al*.[18] conducted a cross-sectional interview of a random sample of 586 Cambodian refugees using CIDI version 2.1 and found higher psychiatric morbidity in older age group. The higher prevalence of psychiatric disorders in the age group 45-54 years could be explained by higher prevalence of depression (which is the most common diagnosis in our study too) in this age group.

In our study, females constituted 50% of the study population and had a higher prevalence of psychiatric disorders than males (37.3% in females and 32% in males). Similarly, higher prevalence of psychiatric disorders was found in Guatemalan refugees.[19] Our findings are also in line with Mollica *et al*.[20] who studied one adult from each of 573 Bosnian refugee camp families using Harvard Trauma Questionnaire and Hopkins-25 and found higher morbidity in females as compared to male refugees.

The lowest psychiatric morbidity was found in post graduates i.e. 25%, which is consistent with others studies.[20,21] In our study, presence of psychiatric disorders was highest in the unemployed and least in self employed, which is also in line with the literature on Cambodian and Afghan refugees.[18,21]

In our study, psychiatric morbidity was more in migrant population than in controls. Similar results have also been observed earlier by Edvard Hauff and Per Vaglum[22] who studied a group of Vietnamese boat refugees on their arrival in Norway and three years later using self rated psychological distress (SCL-90-R). They found that one-third of subjects had a psychiatric disorder. Westermeyer *et al*.[23] assessed most Hmong refugees in Minnesota using two self rated scales (Zung scale for depression and 90 item symptom checklists) where 44% of subjects had psychiatric morbidity.[23] These findings suggest that migration is a stressful event and thus brings out psychiatric morbidity in the predisposed population. Furthermore, the migrant population usually comes from conflict ridden zones already having higher prevalence of disorders such as PTSD, depression and other mental health problems because of traumatic stress, thus, adding to their psychiatric morbidity.

Even though our study clearly showed the higher prevalence of psychiatric disorders in migrant population, this morbidity was reflected only by some of the disorders viz., MDE, PTSD and generalized anxiety disorder (GAD).

In our study, MDE was the most common psychiatric disorder in both migrants as well as controls. Similar results were found in Vietnamese refugees,[22] Cambodian refugees[18] and in Bosnian refugees.[20]

Of the 600 migrants studied, 6.83% (n = 41) had a diagnosis of PTSD current while only 2.5% (n = 5) of the controls had diagnosis of PTSD current. Some studies have reported very high prevalence of PTSD in refugees like the one conducted by Marshall *et al*.[18] on Cambodian refugees two decades after escaping homeland in whom the prevalence of 62% was found. There are other studies where the prevalence rates of PTSD were low as seen by Steel *et al*.[24] in the Vietnamese refugees settled in Australia in whom PTSD rates were 1.5% which were only slightly more than host Australian population (1.4%).

It will be interesting here to compare the rates of PTSD of Kashmiri migrants with the Kashmiri population which did not migrate. According to studies conducted by Margoob *et al*. on Kashmiri population, the lifetime prevalence of PTSD was 15.9%[25] while the current PTSD rates were 7.27% in them.[26]

Nearly 14% (n = 83) of the subjects had features of GAD current while the rate of GAD current in control was 7.5% (n = 15). Similar findings have been reported in Afghan refugees.[21] Studies on Guatemalan refugees have reported the prevalence of GAD to be much higher (54.4%),[19] while studies on Vietnamese refugees have reported very low rates of GAD (0.7%), which were even lower than the host population (2.7%).[24] The migrants are at greater risk of developing generalized anxiety disorder due to problems adjusting to a new culture, feeling of inferiority, alienation and breakdown of social fabric of the migrants.

Alcohol dependence was significantly more common in controls than Kashmiri migrants. About 3.5% (n = 21) of the migrants had alcohol dependence current while 9.5% of the control (host population of Jammu) had diagnoses of alcohol dependence current. This is probably because alcohol is viewed as less socially acceptable in Kashmiri community than in Jammu community, which is also supported by earlier studies by Margoob *et al*. in Kashmiri population.[27] However, interestingly, similar results were also seen by Marshall *et al*. in Cambodian refugees in whom the rates of alcohol dependence were only 4%, which were much lower than that seen in the host U.S population[18] and by Steel *et al*. who found that prevalence of alcohol use disorders was 1.1% in Vietnamese refugees while it was 6.7% in host Australian population.[24]

Low rates of other psychiatric disorders were found in Kashmiri migrants. Eitinger[28] reported high rates of psychosis in migrants to Norway after word war-II. Sanua.[29] found high rates of schizophrenia in immigrants than non immigrants. Afghan refugees demonstrated low rates of manic disorder (0.2%), hypomania episode (0.1%), panic disorder current (0.1%), obsessive-compulsive disorder (OCD) current (0.2%), substance dependence (9.9%), psychotic disorder life time (4.2%), psychotic disorder current (0.1%).[21] Similarly, Vietnamese refugees living in Australia evidenced low rates of panic / agoraphobia (0.6%) social phobia (0.3%), OCD (0.5%), Dysthymia (1.0%), Drug use disorder (0.5%).[24]

### Limitations and suggestions

This study was conducted in migrant camps at one locality of Jammu. It does not necessarily reflect the psychiatric morbidity in the whole migrant population. In this study, only the psychiatric problems of the migrants have been studied while the effect of migration on their cultural and social life has not been explored. Therefore, putative variables contributing to high psychiatric morbidity in migrants could not be established.

Improving the socio-economic status of the migrants, providing effective psychiatric services within the camps, and improving overall psychiatric services in the region of Jammu could be some of the helpful measures.
